# Efficacy and safety of weekly versus triweekly cisplatin treatment concomitant with radiotherapy for locally advanced nasopharyngeal carcinoma: A systematic review and pooled analysis

**DOI:** 10.3389/fphar.2022.999027

**Published:** 2023-01-05

**Authors:** Xin Tian, Qiuxia Zhu, Zhenyong Zhang

**Affiliations:** Department of Oncology, Shengjing Hospital of China Medical University, Shenyang, China

**Keywords:** weekly, triweekly, cisplatin, radiotherapy, nasopharyngeal carcinoma

## Abstract

**Background:** Most nasopharyngeal carcinoma cases are diagnosed at an advanced stage due to their hidden anatomical structure and atypical clinical symptoms and often require chemoradiotherapy. Here, we present a systematic review and pooled analysis to synthesize existing research on the efficacy and adverse effects of weekly versus triweekly cisplatin chemotherapy concomitant with radiotherapy for locally advanced nasopharyngeal carcinoma (LANPC).

**Methods:** We searched the PubMed, Embase, and Cochrane Library databases from inception to 1 September 2021, for relevant original research articles published in English. The literature search and data extraction were done independently by two investigators. We used random-effects models to provide point estimates [95% confidence interval (CI)] of overall response rate (ORR), overall survival (OS), progression-free survival (PFS), locoregional recurrence-free survival (LRFS), distant metastasis-free survival (DMFS) and the incidence rate of adverse effects (AEs) and with subgroup analysis according to each study type. The primary endpoints were ORR, OS, and PFS; LRFS, DMFS, and grade ≥3 acute AEs were secondary endpoints.

**Results:** In total, 2,305 patients of eight studies were included in this review. We found that patients who were administered cisplatin weekly or triweekly had no differences in ORR, OS, PFS, DMFS, LRFS, severe mucositis, dermatitis, nausea/vomiting or nephrotoxicity. Patients who were administered weekly cisplatin were at a higher risk of hematological toxicity compared with patients who received the chemotherapy triweekly.

**Conclusion:** Our findings suggest that both regimens could be recommended as the standard of care for the chemoradiotherapy treatment of LANPC, the perceived benefit of lower toxicity with weekly cisplatin could not be established.

## 1 Introduction

Most nasopharyngeal carcinoma (NPC) cases are diagnosed at an advanced stage due to the unique anatomical position of tumors and varied clinical manifestations (Torre et al., 2015). Patients with locally advanced nasopharyngeal carcinoma (LANPC) are often treated with radiotherapy and adjuvant chemotherapy to reduce local recurrence and distant metastasis (Li et al., 2019; Li et al., 2019). Common chemotherapy agents that demonstrate efficacy against NPC include platinum compounds (cisplatin and carboplatin), fiuorouracil, and taxanes (docetaxel and paclitaxel). Cisplatin-based regimens have been established as the most effective, and a dose of 100 mg/m^2^ given every 3 weeks is the most common (Tang et al., 2018). However, the majority of patients who undergo chemoradiotherapy will suffer from serious radiotherapy-induced mucosa and skin damage, hematological toxicity, and gastrointestinal reactions (Lee et al., 2010). Adverse effects (AEs) tend to cause patients extreme pain, which leads some patients to renounce their treatments (Chen et al., 2021). Accordingly, minimizing adverse effects during treatment is an important goal for clinical oncologists. In recent years, increasing evidence has indicated that weekly chemotherapy has similar efficacy and lower toxicity than triweekly regimens for many types of cancers (Schuette et al., 2005; Dessai et al., 2016; Tousif et al., 2020). Multiple studies have compared the outcomes of weekly and triweekly cisplatin concurrent chemoradiotherapy (CCRT) in advanced head and neck squamous cell carcinoma (HNSCC) (Espelia et al., 2012; Geiger et al., 2014; Tsan et al., 2012). Most studies have excluded analysis solely of a cohort of patients with nasopharyngeal cancer, as the biology and behavior of this malignancy differ from other HNSCC. A previous meta-analysis was conducted to compare benefits and risks of weekly and triweekly cisplatin schedules across NPC patients during CCRT (Tang et al., 2021). The study concluded that both weekly and triweekly schedules could be recommended to NPC patients. However there were only four retrospective studies eligible in that pooled analysis. Therefore, we performed this pooled analysis which included more retrospective studies and randomized controlled trials (RCTs) to synthesize results from the existing literature to compare the efficacies and adverse effects of the different cisplatin schedules for patients with LANPC.

## 2 Materials and methods

### 2.1 Search strategy

This systematic review and pooled analysis were conducted in accordance with the preferred reporting items for systematic reviews and meta-analyses (PRISMA) guidelines. Two authors (XT and QXZ) independently searched for published studies in PubMed, Embase, and the Cochrane Library from inception to 1 September 2021 and used search terms that were chosen in collaboration with an experienced medical librarian. The search key words “weekly,” “3-weekly,” “triweekly,” “cisplatin,” “nasopharyngeal carcinoma,” “radiotherapy,” “chemoradiotherapy,” “chemoradiation,” and “radiation” were used in both “AND” and “OR” combinations.

### 2.2 Eligibility and exclusion criteria

We included all relevant studies that provided data regarding overall response rate (ORR), overall survival (OS), progression-free survival (PFS), distant metastasis-free survival (DMFS), locoregional recurrence-free survival (LRFS), and AEs in patients with LANPC who underwent definitive chemoradiation with weekly or triweekly cisplatin chemotherapy. The inclusion criteria were as follows: 1) LANPC was diagnosed by histopathological findings; 2) previously untreated and non-distant metastatic cases; 3) patients treated with chemoradiotherapy; 4) a risk ratio (RR) or hazard ratio (HR) with 95% confidence intervals (CIs) was available. Exclusion criteria for the analysis were as follows: 1) The study was a review article, abstract, case report, poster, conference paper, thesis, or book; 2) studies that included fewer than 30 patients; 3) the study was single armed; 4) the study used other chemotherapy drugs; We brought all the searched results from the three electronic data-bases above into EndNote (Thomson Reuters, PA, US). Only publications written in English were included. Publications with duplicate data were excluded. Articles were initially screened by title and abstract, and then full-text articles were assessed to identify eligible studies. Two reviewers (XT and QXZ) independently evaluated all of the included studies. Disagreements about inclusion and exclusion were resolved by discussion between the two authors or by consulting a third senior researcher (ZYZ).

### 2.3 Data selection, extraction, and quality assessment

Two reviewers independently reviewed the full texts of all relevant research papers. Data were extracted into a designated worksheet and included the following: first authors; country; year of publication; study design type; number of patients; dosage of chemotherapy and radiotherapy; hazard ratios (HR) and 95% CIs for OS, PFS, DMFS, and LRFS; and risk ratios (RR) and 95% CIs for ORR and AEs. Data were extracted independently by two investigators (XT and QXZ), and disagreements were resolved by consensus or through discussion with a third reviewer (ZYZ). The risk of bias of each RCT was assessed according to the Cochrane collaboration network’s bias risk assessment criteria. The risk of bias in each retrospective study was assessed according to the Newcastle-Ottawa scale (NOS), which included the selection process, comparability, and outcome of this study. Studies with the Cochrane collaboration network with low to moderate risk of bias or NOS scores ≥6 were considered high-quality and included in this meta-analysis.

### 2.4 Data synthesis and statistical analysis

Data were analyzed using STATA (version 12.0, Stata Corporation, College Station, TX, United States). The primary outcomes were ORR, OS, and PFS; the secondary endpoints were LRFS, DMFS, and grade ≥3 AEs. The survival outcomes related to PFS, OS, LRFS, and DMFS were expressed as the HR with a 95% CI while the outcomes related to ORR and AEs were expressed as the RR with a 95% CI. We assessed heterogeneity using the Cochran Q-statistic and the I^2^ statistic. Estimates with a *p*-value lower than .05 for the Q-statistic and I^2^ of 50% or greater were considered to have moderate heterogeneity. As the included studies differed regarding their design and patients’ baselines, pooled analyses were performed using a random-effects model. Subgroup analyses were conducted according to different study types. Additionally, publication bias was assessed using funnel plots and Egger’s and Begg’s tests when *n* > 5 studies.

## 3 Results

### 3.1 Study selection and patient characteristics

**FIGURE 1:**
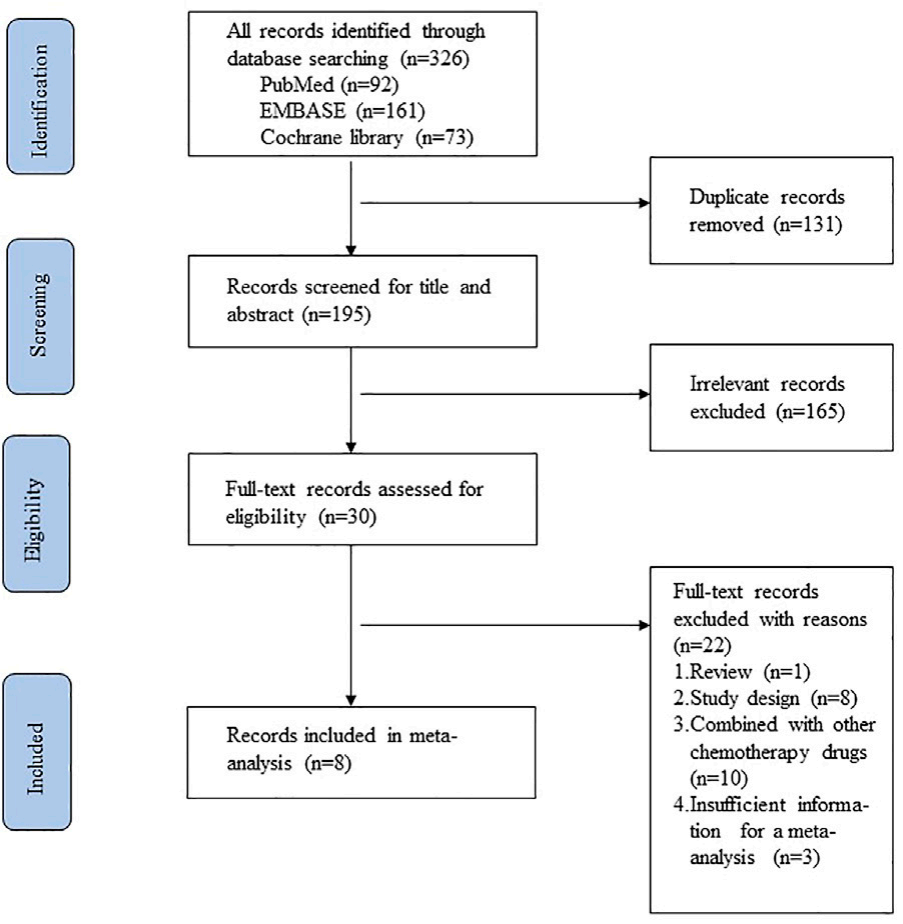
Selection process for eligible research.

According to the PRISMA flow chart, the selection process for eligible research is illustrated in Figure 1. A total of 326 articles were identified by searching the databases, comprising 92 from Pubmed, 161 from Embase, and 73 from Cochrane Library. We excluded 131 duplicate studies. Next, 165 irrelevant studies were excluded upon reviewing the titles and abstracts. The full texts of 121 the 30 remaining studies were reviewed, and eight studies that met the inclusion criteria were ultimately included. Characteristics of the selected studies are summarized in Table 1. Six retrospective studies (Jagdis et al., 2014; Tao et al., 2014; Meng et al., 2018; Zhu et al., 2018; Wang et al., 2019; Gundog et al., 2020) and two RCTs (Lee et al., 2016; Xia et al., 2021) were included in the analysis. The RCTs included one phase II and one phase III clinical trials. All of the eight studies included a total of 2,305 patients with LANPC. Of these, 890 received weekly cisplatin chemotherapy + radiotherapy and 1,415 received triweekly cisplatin chemotherapy + radiotherapy. A total of four studies reported the outcomes of ORR; seven studies reported the outcomes of survival; and eight studies reported the results of AES.

**TABLE 1:** Main characteristics of the included studies.

First author	Year	Country	Study design	Groups	No. patients	Dosage	Radiotherapy	Quality
Tao	2014	China	Retro	Weekly	73	30–40 mg/m^2^	IMRT	High quality
				Triweekly	81	80 mg/m^2^		
Jagdis	2014	British	Retro	Weekly	45	40 mg/m^2^	3D-CRT/IMRT	High quality
				Triweekly	28	100 mg/m^2^		
Zhu	2018	China	Retro	Weekly	225	40 mg/m^2^	IMRT	High quality
				Triweekly	634	100 mg/m^2^		
Meng	2018	China	Retro	Weekly	90	30–40 mg/m^2^	IMRT	High quality
				Triweekly	90	80 mg/m^2^		
Wang	2019	China	Retro	Weekly	93	30–40 mg/m^2^	IMRT	High quality
				Triweekly	229	80–100 mg/m^2^		
Gundog	2020	Türkiye	Retro	Weekly	61	50 mg/m^2^	2D/3D-CRT IMRT	High quality
				Triweekly	37	100 mg/m^2^		
Lee	2016	Korea	RCT Ⅱ	Weekly	53	40 mg/m^2^	3D-CRT/IMRT	Low risk
				Triweekly	56	100 mg/m^2^		
Xia	2021	China	RCT Ⅲ	Weekly	250	40 mg/m^2^	IMRT	Low risk
				Triweekly	260	100 mg/m^2^		

Retro, retrospective; IMRT, intensity modulated radiation therapy; 2D/3D-CRT, Two/Three dimensional conformal radiation therapy.

### 3.2 Risk of bias of included studies

The risk of bias of the two RCTs in our studies was assessed according to the Cochrane collaboration network’s bias risk assessment criteria as having a low risk of bias (Table 1). The risk of bias of six retrospective studies was assessed with NOS scores ≥6 which were generally considered high quality.

### 3.3 Clinical efficacy

**FIGURE 2:**
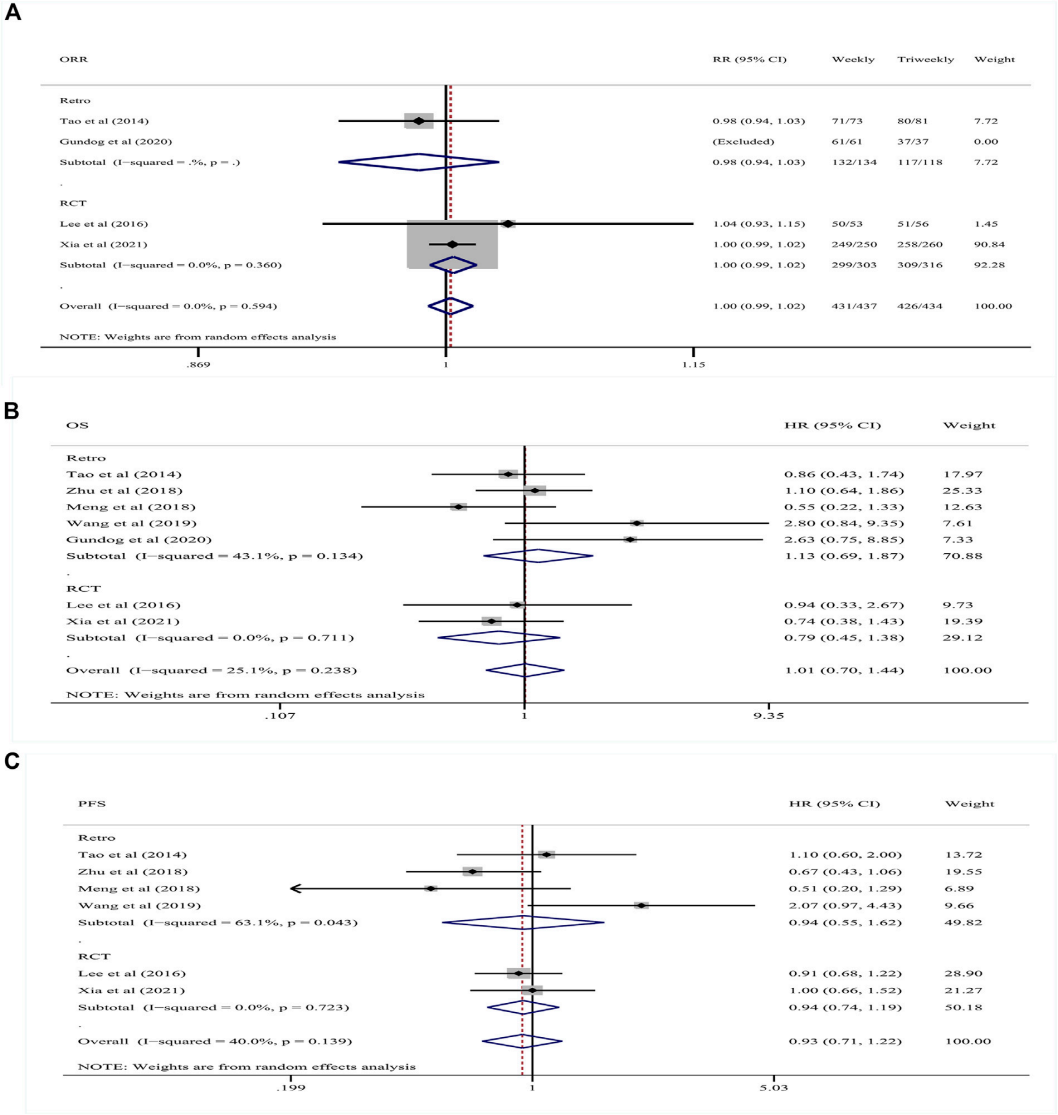
Forest plots of the comparison of **(A)** ORR; **(B)** OS, and **(C)** PFS between patients administered cisplatin weekly or triweekly. ORR, objective response rate; OS, overall survival; PFS, progression-free survival.

Two retrospective study and two RCTs reported data on ORR. The two retrospective studies included 134 patients in the weekly group and 118 patients in the triweekly group, while the two RCTs included 303 patients in the weekly group and 316 patients in the triweekly group. As the included studies differed regarding the study design and patient baselines, a random-effects model was used. The pooled RR indicated no difference in ORR between patients who were administered cisplatin weekly or triweekly in the retrospective studies (RR: .98; 95% CI: .94–1.03, *p* = .509) 145 and the RCTs (RR: 1.00; 95% CI: .99–1.02; *p* = .531; I^2^ = 0%) nor in all studies (RR: 1.00; 95% CI 146 .99–1.02; *p* = .676; I^2^ = 0%) (Figure 2A).

### 3.4 Survival

#### 3.4.1 OS and PFS

Five retrospective studies and two RCTs reported data on OS and PFS. The five retrospective studies included 542 patients in the weekly group and 1,071 patients in the triweekly group while the two RCTs included 303 patients in the weekly group and 316 patients in the triweekly group. A random-effects model was used because of the different types of studies included. The pooled HR of OS in all studies was 1.01 (95% CI .70–1.44, *p* = .976; I^2^ = 25.1%), which indicated no difference in OS between the weekly and triweekly groups. In subgroup analyses according to the different study designs, the pooled HR was 1.13 (95% CI .69–1.87, *p* = .621; I^2^ = 43.1%) in the retrospective studies and .79 (95% CI .45–1.38, *p* = .412; I^2^ = 0%) in the RCTs and did not differ between the cisplatin treatment regimens. The pooled HR in all studies of PFS was .93 (95% CI .71–1.22, *p* = .616; I^2^ = 40%), which indicated no difference in PFS between patients treated weekly versus triweekly. In subgroup analyses according to different study designs, the pooled HR was .94 (95% CI .55–1.22, *p* = .823; I^2^ = 63.1%) in the retrospective studies and .94 (95% CI .74–1.19, *p* = .613; I^2^ = 0%) in the RCTs, and were not different between the treatment regimens regardless of the study designs (Figures 2B, C).

#### 3.4.2 DMFS and LRFS

**FIGURE 3:**
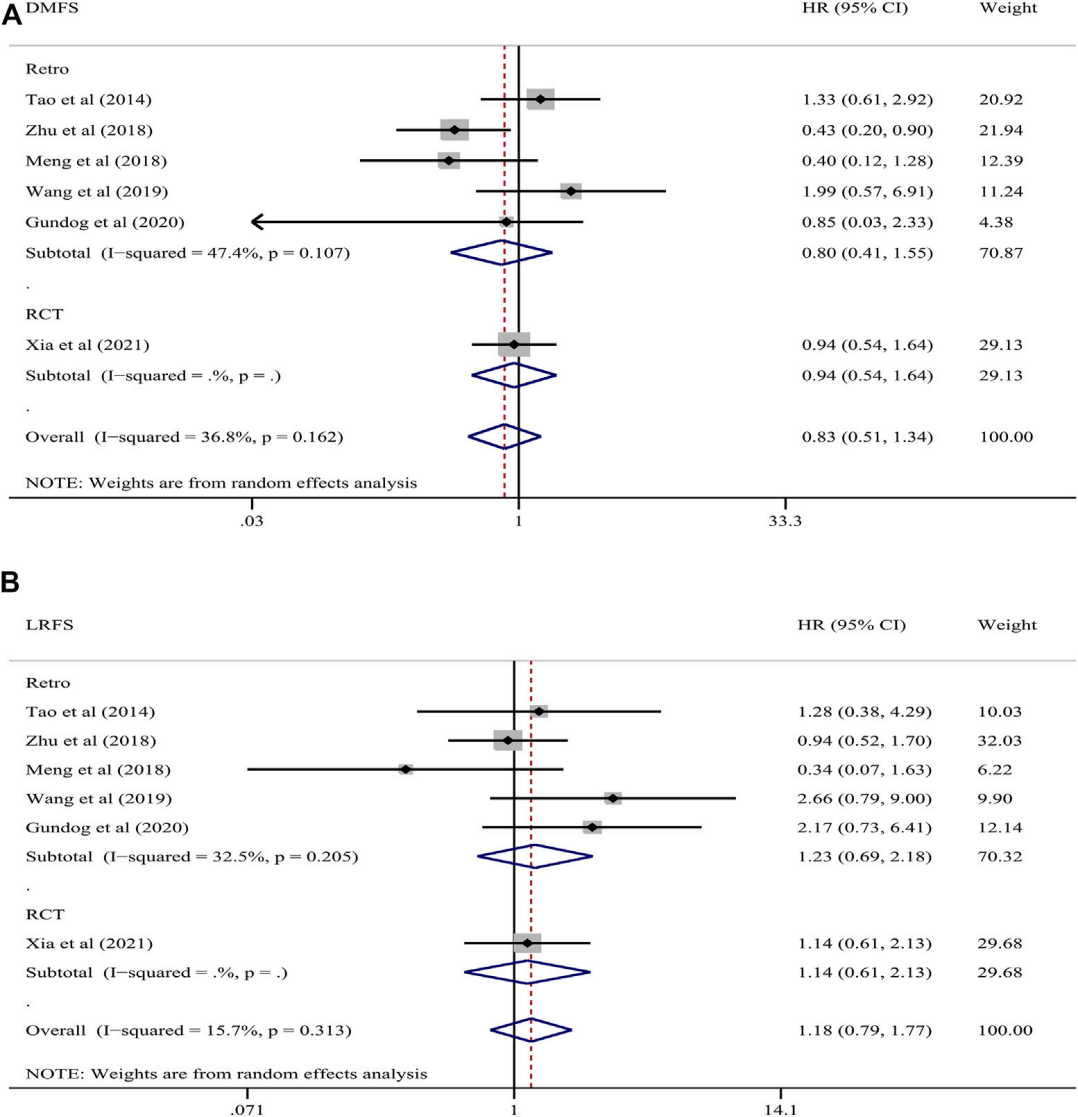
Forest plots of the comparison of **(A)** DMFS and **(B)** LRFS between patients administered cisplatin weekly or triweekly. DMFS, distant metastasis-free survival; LRFS, locoregional recurrence-free survival.

Five retrospective studies and one RCT reported data on DMFS and LRFS. The five retrospective studies included 542 patients in the weekly group and 1,071 patients in the triweekly group while the RCT included 250 patients in the weekly group and 260 patients in the triweekly group. A random-effects model was used given that different types of studies were analyzed. The pooled HR of DMFS in all studies was .83 (95% CI .51–1.34; *p* = .442; I^2^ = 36.8%), which indicated that there was no difference in DMFS between the cisplatin regiments. In subgroup analyses according to the different types of study design, the pooled HR was .80 (95% CI .41–1.55; *p* = .504; I^2^ = 47.4%) in the retrospective studies and .94 (95% CI 0. 54–1.64; *p* = .827) in the RCT, which indicated no difference between weekly versus triweekly treatment. The pooled HR of LRFS in all studies was 1.18 (95% CI .79–1.77; *p* = .414; I^2^ = 15.7%), which indicated no difference in LRFS between the treatment groups. In subgroup analyses according to the different study designs, the pooled HR was 1.23 (95% CI .69–2.18; *p* = .478; I^2^ = 32.5%) in the retrospective studies and 1.14 (95% CI .61–2.13; *p* = .681) in the RCT and did not differ between the two treatment groups, regardless of the study type (Figure 3).

### 3.5 Adverse reactions

**FIGURE 4:**
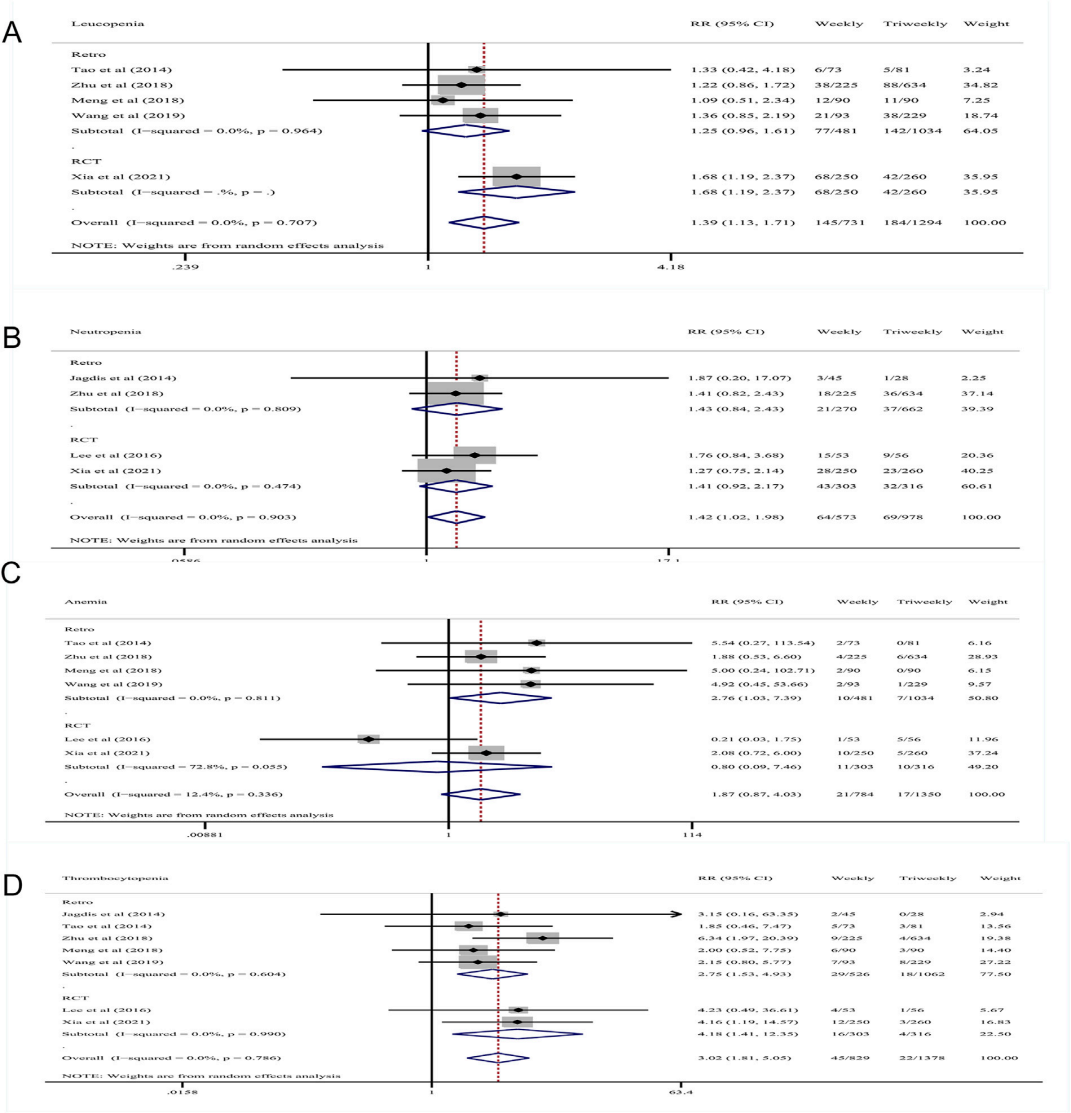
Forest plots of the RRs of Severe Hematological AEs **(A)** grade 3–4 leukopenia; **(B)** grade 3–4 neutropenia; **(C)** grade 3–4 anemia; **(D)** grade 3–4 thrombocytopenia. RR, risk ratios; AEs, adverse effects.

**FIGURE 5:**
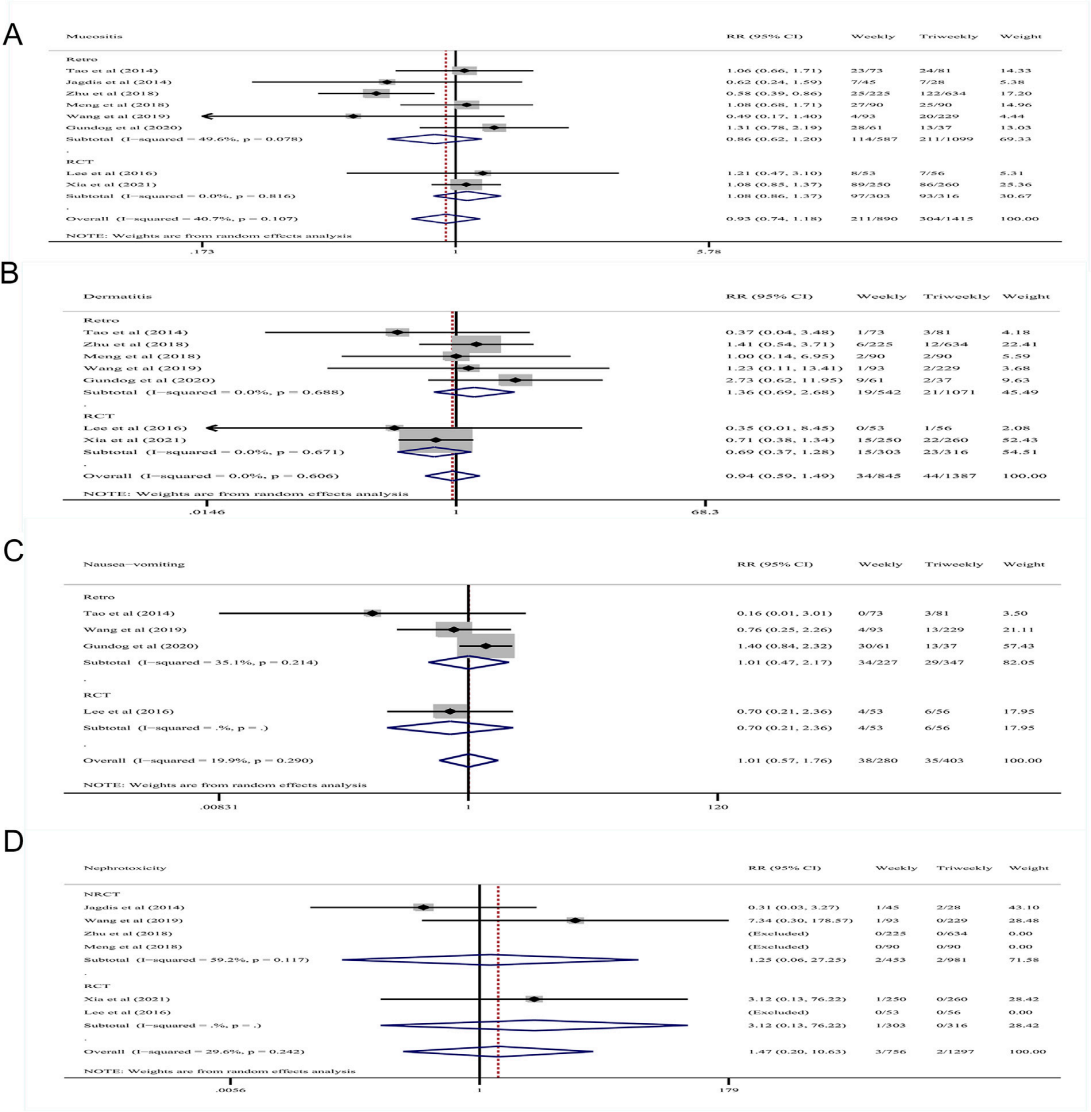
Forest plots of the RRs of Severe Non-hematological AEs **(A)** grade 3–4 mucositis; **(B)** grade 3–4 dermatitis; **(C)** grade 3–4 nausea/vomiting; **(D)** grade 3–4 nephrotoxicity. RR, risk ratios; AEs, adverse effects.

AEs (grade ≥ 3) were used to evaluate the safety of weekly and triweekly cisplatin chemotherapy concurrent with radiotherapy in treatment of LANPC. Five retrospective studies and two RCTs reported data on hematological AEs, while six retrospective studies and two RCTs provided data on non-hematological AEs. The pooled RRs of severe AEs (grade ≥ 3) are displayed in Figure 4 and Figure 5.

#### 3.5.1 Severe hematological AEs

Five retrospective studies including 526 patients in the weekly group and 1,062 patients in the triweekly group, as well as two RCTs including 303 patients in the weekly group and 316 patients in the triweekly group reported data on hematological AEs. The pooled RR of grade ≥ 3 thrombocytopenia indicated that patients treated weekly were at a higher risk in both the retrospective studies (RR: 2.75; 95% CI: 1.53–4.93; *p* = .001; I^2^ = 0%) and RCTs (RR: 4.18; 95% CI: 1.41–12.35; *p* = .010; I^2^ = 0%), as well as in all studies (RR: 3.02; 95% CI 1.81–5.05; *p* = .000; I^2^ = 0%). The pooled RR of grade ≥ 3 leukopenia indicated that weekly treatment was associated with greater risk in RCTs (RR: 1.68; 95% CI: 1.19–2.37; *p* = .003) and in all studies (RR: 1.39; 95% CI 1.13–1.71; *p* = .002; I^2^ = 0%). The pooled RR of grade ≥ 3 neutropenia indicated that patients who had been treated with cisplatin weekly had a higher risk in all of the studies (RR: 1.42; 95% CI 1.02–1.98; *p* = .038; I^2^ = 0%). The pooled RR of grade ≥ 3 anemia indicated that patients who had been treated weekly had a higher risk in retrospective studies (RR: 2.76; 95% CI 1.03–7.39; *p* = .044; I^2^ = 0%) (Figure 4).

#### 3.5.2 Severe non-hematological AEs

The most common non-hematological AEs were mucositis, dermatitis, nausea/vomiting and nephrotoxicity. Six retrospective studies, including 587 patients in the weekly group and 1,099 patients in the triweekly group, while two RCTs including 303 patients in the weekly group and 316 patients in the triweekly group reported data on non-hematological AEs. The pooled RRs for grade ≥ 3 mucositis (RR = .93, 95% CI .74–1.18; *p* = .575; I^2^ = 40.7%), dermatitis (RR = .94, 95% CI .59–1.49; *p* = .790; I^2^ = 0%), nausea/vomiting (RR = 1.01, 95% CI .57–1.76; *p* = .980; I^2^ = 19.9%), and nephrotoxicity (RR = 1.47, 95% CI .20–10.63; *p* = .700; I^2^ = 29.6%) indicated no differences in these AEs between patients treated with either cisplatin regimen.

In further subgroup analysis according to the study type, pooled RRs of grade ≥ 3 mucositis, dermatitis, nausea/vomiting and nephrotoxicity also indicated no differences in these AEs between the treatment groups regardless of the study type (Figure 5).

### 3.6 Publication bias

**FIGURE 6:**
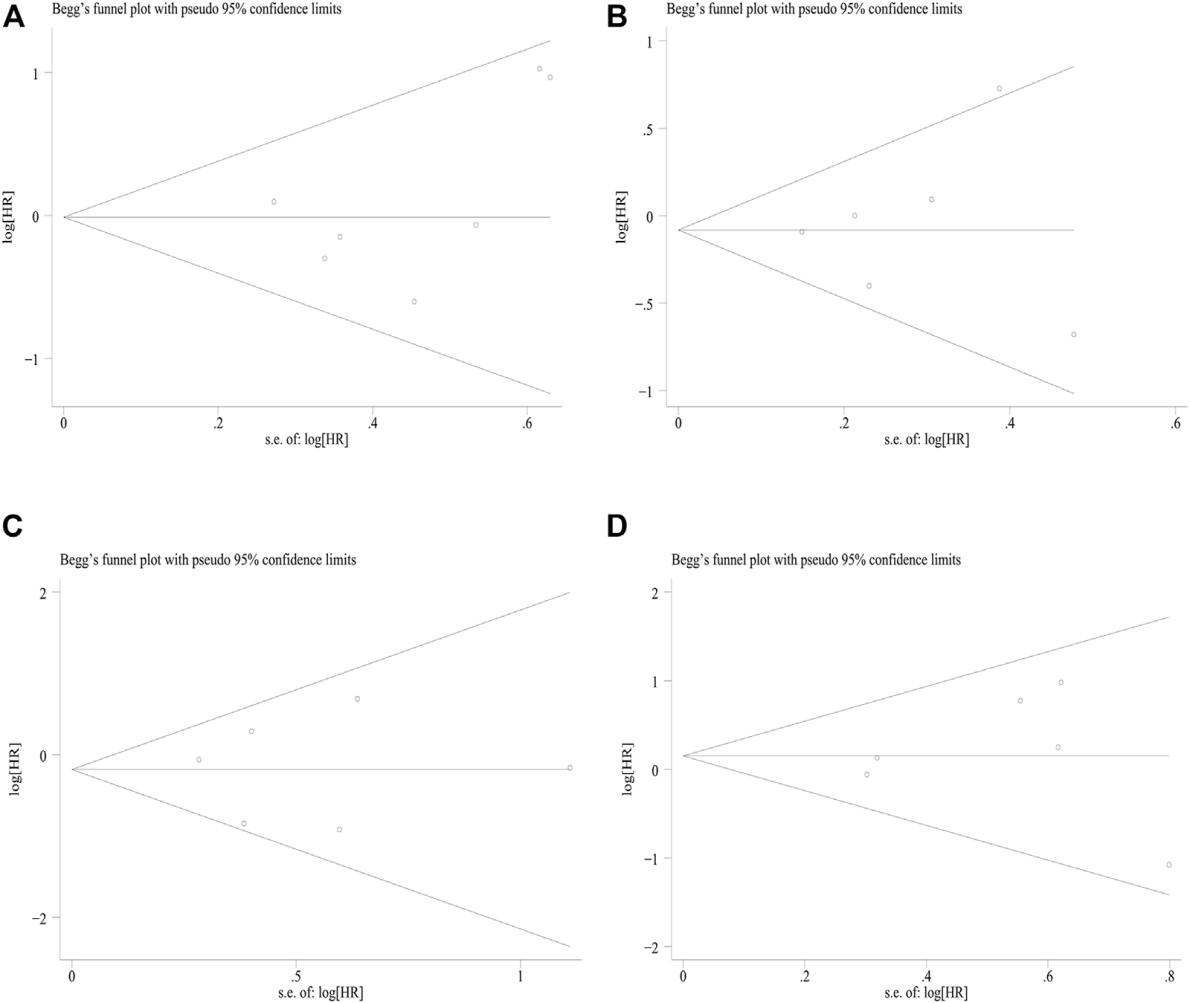
Publication bias assessed by funnel plot of **(A)** OS; **(B)** PFS; **(C)** DMFS; **(D)** LRFS. OS, overall survival; PFS, progression-free survival; DMFS, distant metastasis-free survival; LRFS, locoregional recurrence-free survival.

Begg’s and Egger’s tests were conducted to assess the publication biases among survival studies. The results of the OS tests were z = .90 (*p* = .368) and t = 1.15 (*p* = .301); the results of the PFS tests were z = .38 (*p* = .707) and t = .28 (*p* = .797); the results of the DMFS tests were z = .38 (*p* = .707) and t = .01 (*p* = .994); the results of the LRFS tests were z = .38 (*p* = .707) and t = .31 (*p* = .772). Thus, there was no evidence of publication bias in the meta-analyses of OS, PFS, DMFS, nor LRFS. A funnel plot of Begg’s test is presented in Figure 6.

## 4 Discussion

This study analyzed the existing research results concerning weekly and triweekly cisplatin regimens concomitant with radiotherapy in patients with LANPC. In the pooled analyses, ORR, OS, PFS, DMFS, and LRFS were similar in both arms. Severe mucositis, dermatitis, nausea/vomiting and nephrotoxicity were similar in both arms. However, patients in the weekly group had an increased risks of grades ≥3 hematological toxicity.

Chemoradiotherapy is the standard treatment for LANPC (Chan et al., 2005). A large number of clinical studies have confirmed the role of chemoradiotherapy in improving both the local control rate of tumors and causing an improvement in overall survival (Chitapanarux et al., 2007; Wu et al., 2013; Blanchard et al., 2015). Synchronous chemoradiotherapy has the following advantages: 1) a synergistic effect, as chemotherapy drugs can improve the sensitivity of tumor cells to radiotherapy, and radiotherapy can also enhance the cytotoxicity of chemotherapy drugs; 2) in theory, chemotherapy can also eliminate small occult metastases and reduce the rate of distant metastasis.

Among all chemotherapy drugs used with radiation therapy for LANPC, cisplatin is the most common (Li et al., 2019). However, chemoradiation is associated with more AEs, which leads to lower compliance rates among patients compared with those who receive radiotherapy only (Lee et al., 2010; Tang et al., 2018; Jiang et al., 2019). One major challenge faced by clinicians is how to reduce the adverse reactions in patients undergoing chemoradiotherapy while ensuring the maximum possible curative effects. Previous studies have shown that weekly cisplatin has good anti-tumor efficacy and is welltolerized in esophageal squamous cell carcinoma, ovarian cancer, and other malignant tumors (Verborg et al., 2008; Caro et al., 2016; Mazzola et al., 2017). The high toxicity of cisplatin led to the assumption that a moderate dose given weekly may be desirable. Studies on the pharmacokinetics of cisplatin have shown the possible superiority of a weekly schedule, which can provide a radiosensitizing effect and reduce toxicity without compromising efficacy (Kurihara et al., 1996; Boulikas et al., 2003).

Studies on weekly and triweekly cisplatin regimens of chemotherapy coinciding with radiation have been reported on HNSCC (Uygun et al., 2009; Kose et al., 2011; Szturz et al., 2017). However, most of them excluded population of nasopharyngeal cancer, there is little known about the comparative efficacies of these treatment regimens for nasopharyngeal carcinoma, which was the focus of our study. However, there is a paucity of data from RCTs due to the low incidence of nasopharyngeal cancer.

In our study, only two qualified RCTs were included, and the remaining six were NRCTs. Well-designed NRCTs are not necessarily more biased than RCTs, as NRCTs often have larger sample sizes and establish more reliable evidence of real world. The six retrospective studies included had sufficient sample sizes, and the literature quality was evaluated to meet the NOS standard. Additionally, Begg’s and Egger’s tests found no evidence of publication bias in the studies. However, in the retrospective studies the chemotherapy choice and assessment of disease progression were decided by physicians, which increased the risk of bias. Thus, pooled analyses were performed using a more conservative random-effects model. In the combined analysis of the final data, we combined the data of two RCTs, six retrospective studies, and all the studies. The results for ORR, survival, and AEs were essentially the same, regardless of the study design type, and there were no significantly different results between the different study types. Therefore, our study is of certain clinical significance.

In our study, the combined analysis of the pooled RRs of ORR, OS, PFS, DMFS, and LRFS were similar in weekly and triweekly groups. The similar clinical efficacy of this two different dosing regimens may have resulted from several reasons. First, cisplatin is chemotherapeutic drug that exert its anticancer effect by causing cross-linking of DNA leading to inhibition of its synthesis and induction of apoptosis. It has been suggested that a cumulative dose of 200 mg/m^2^ needs to be reached for a therapeutic benefit in cisplatin studies (Geeta et al., 2006). In our study, the cumulative doses of cisplatin in patients treated weekly or triweekly were nearly the same (Table 2), and this possibly contributed to the similarities in efficacy and survival. Second, NPC is a malignant tumor that is highly sensitive to radiotherapy. In recent years, radiotherapy equipment and technology are continuously improving, and intensity modulated radiotherapy (IMRT) has been widely used in the treatment of NPC. Compared with two-dimensional therapy, IMRT has a better dose distribution and can improve the local regional control rate. Better local control rates may improve OS. In most of our included studies, concurrent radiotherapy used IMRT, and we believe that the use of this technique played a significant role in the treatment effect of patients in the two groups. Finally, although pooled analyses were performed using a more conservative random-effects model, a selection bias of the included retrospective studies remained, in which patients with high T and N stage were likely to be assigned in triweekly group (Jagdis et al., 2014; Zhu et al., 2018; Wang et al., 2019; Gundog et al., 2020).

**TABLE 2:** Summary of mean cumulative dose of cisplatin.

First author	Year	Groups	Mean cisplatin dose	*p*-value
Tao	2014	Weekly	180 mg/m^2^	.10
		Triweekly	160 mg/m^2^	
Jagdis	2014	Weekly	230 mg/m^2^	—
		Triweekly	249 mg/m^2^	
Zhu	2018	Weekly	229.2 mg/m^2^	—
		Triweekly	228.0 mg/m^2^	
Meng	2018	Weekly	171.0 mg/m^2^	.426
		Triweekly	168.2 mg/m^2^	
Wang	2019	Weekly	190.54 mg/m^2^	.062
		Triweekly	202.79 mg/m^2^	
Lee	2016	Weekly	248.9 mg/m^2^	.433
		Triweekly	256.6 mg/m^2^	
Xia	2021	Weekly	220 mg/m^2^	—
		Triweekly	200 mg/m^2^	

When we evaluated the efficiency and safety of the two regimens, it was necessary to take into consideration the most common AEs. In the case of similar treatment results, adverse reactions are more concerning for both clinicians and patients. One of the main reasons of shifting from a triweekly to weekly schedule is to reduce adverse reactions. In our study, the weekly group was at a higher risk of severe hematological toxicity especially of thrombocytopenia and leukopenia. This may be explained by the selection bias of retrospective studies, in which those who had older age, (Jagdis et al., 2014; Tao et al., 2014; Meng et al., 2018; Zhu et al., 2018; Wang et al., 2019; Gundog et al., 2020) and poor physical condition (Jagdis et al., 2014; Zhu et al., 2018) were likely to be administered weekly cisplatin. These patients are less tolerant of chemoradiotherapy and have a higher incidence of adverse reactions. Regarding non-hematological AEs, the pooled RRs for grade ≥ 3 mucositis, dermatitis in our results indicated no differences between patients treated with either cisplatin regimen. This may have been due to the included studies using IMRT as the concurrent radiotherapy, which can better protect the surrounding normal tissue and organs. Therefore, more RCTs with larger sample size are needed to confirm these results. Triweekly regimen can be confidently adopted without compromising the outcomes and toxicity profiles until the release of more clinical trials with larger sample size and longer follow-up durations. Our results may provide clinical treatment guidelines in most situations and useful information for the design of future clinical studies. Several limitations of our study should be noted. The major limitation of this systematic analysis is the inclusion of only two RCTs, while the remaining six studies were NRCTs due to the low incidence of LANPC. As NRCT studies may have a selection bias, a large number of multicenter RCTs with larger sample sizes should be conducted to confirm the efficacies of each therapeutic approach and AEs. Second, there was moderately heterogeneity in several comparisons (PFS, AEs), which would have an impact on the stability of these results. Third, we only considered high-quality articles published in English, which may have introduced a language bias.

## 5 Conclusion

This study found both weekly and triweekly cisplatin regimens have similar clinical efficacies in ORR, OS, PFS, DMFS, and LRFS for LANPC. Acute treatment-related toxicities were also similar, with the exception of hematological toxicity favoring the triweekly regimen. While both regimens could be recommended as the standard of care, the perceived benefit of lower toxicity with weekly cisplatin could not be established.

## Data Availability

The original contributions presented in the study are included in the article/supplementary material, further inquiries can be directed to the corresponding author.
